# Anticoagulation therapy for pulmonary embolism involving a myxoma mimicking, giant type C thrombus: A case report

**DOI:** 10.7555/JBR.36.20220118

**Published:** 2022-07-28

**Authors:** Yinhe Feng, Yubin Wang, Xiaolong Li, Hui Mao

**Affiliations:** 1 Department of Respiratory and Critical Care Medicine, People's Hospital of Deyang City, Affiliated Hospital of Chengdu College of Medicine, Deyang, Sichuan 618000, China; 2 Department of Respiratory and Critical Care Medicine, West China Hospital, Sichuan University, Chengdu, Sichuan 610041, China

**Keywords:** pulmonary embolism, intracardiac thrombus, atrial myxoma, anticoagulant

## Abstract

Right heart thrombus (RHTh) with concurrent acute pulmonary embolism (PE) is rare and can seriously destabilize hemodynamics, leading to an emergency situation with high mortality. Diagnosis and treatment of RHTh with acute PE are not yet standardized. There are few reports of acute PE concurrent with RHTh and even less is known about patients with a right heart mural thrombus. For physicians, the diagnostic choice and treatment of these patients are particularly difficult due to the lack of knowledge. Here, we report a rare case of partial mural RHTh (type C RHTh) with acute PE. The mural mass in the right heart was initially diagnosed as atrial myxoma according to transthoracic echocardiography (TTE), and both pulmonary embolus and the mural mass were completely absorbed after administering Rivaroxiban. This case suggests that TTE alone is insufficient to identify and diagnoses a right heart mural mass such as this. However, novel oral anticoagulants may be effective at alleviating PE with type C RHTh.

## Introduction

Venous thromboembolism (VTE), which can present clinically as pulmonary embolism (PE) and/or deep vein thrombosis (DVT), is the third most frequent acute cardiovascular syndrome worldwide, after myocardial infarction and cerebrovascular incidents such as stroke^[1]^. The global annual incidence of PE ranges from 40 to 120 cases per 100 000, in the general population^[2]^. PE causes approximately 300 000 deaths per year in the US alone, making it one of the leading causes of cardiovascular mortality^[2]^. However, in rare instances, acute PE cases present with right heart thrombus (RHTh). With a prevalence of 4.8% this situation is considered an absolute emergency and is associated with high mortality^[3]^.

While a right heart floating thrombus is relatively straightforward and can be differentiated from primary cardiac diseases such as atrial myxoma using transthoracic echocardiography (TTE), diagnosis of right heart mural thrombus is more challenging.

The optimal treatment for concurrent right heart floating thrombus and PE has not been standardized. This is due, at least in part, to the low prevalence which makes large scale randomized controlled trials difficult and funding. Studies have somewhat inconsistently recommended anticoagulation, thrombolytic therapy, surgical removal and percutaneous thrombus retrieval^[4]^. Even less is known about patients with a right heart mural thrombus with PE. Here, we report an extremely rare case of right heart giant, partial mural thrombus concurrent with PE. It is hoped that this report may improve differential diagnosis and reduce misdiagnosis of right heart masses, thus providing a reference standard for the treatment.

Informed consent was requested and obtained from our patient. All procedures followed the ethical standards of the People's Hospital, Deyang City and in accordance with the Helsinki Declaration.

## Case report

A 76-year-old man with a history of essential arterial hypertension was admitted to hospital after experiencing blood in his sputum followed by eight days of progressive dyspnea. This patient was on a regular course of Lercanidipine (10 mg) and Valsartan (160 mg) to be ingested once daily. Upon presentation this man had no complaints of chill, fever, hot flushes, night sweats or chest pains. He did not report having been exposed to harmful environmental agents which might have been causal. Physical examination revealed a temperature of 36.6 ℃, heart rate of 108 beats/minute, respiratory rate of 26 cycles/minute, blood pressure of 125/72 mmHg, and oxygen saturation of 89% in ambient air. This patient had unremarkable signs, except bilateral basal fine moist rales and lower limb swelling. The patient showed no signs of right heart failure.

**Figure 1:**
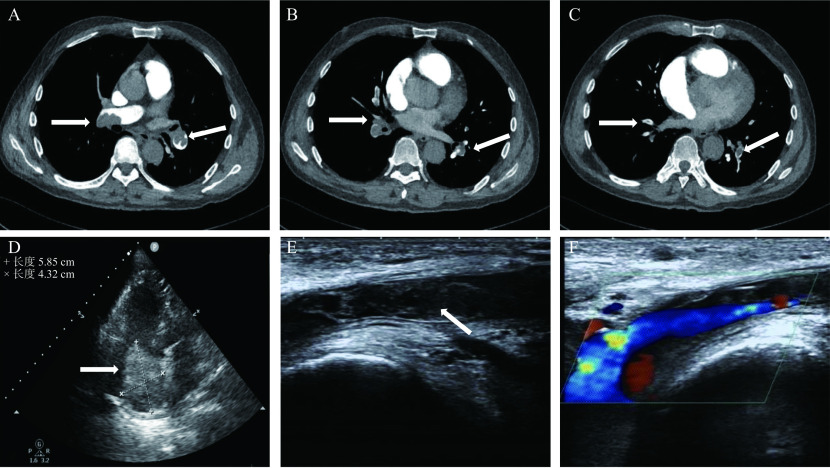
Initial imaging of the patient.

Additionally, this patient had a white blood cell count of 4.3×10^9^/L, neutrophil percentage (69.9%), hemoglobin (115 g/L), C-reactive protein (36.7 mg/L), N-terminal pro-brain natriuretic peptide (525 pg/mL), and D-dimer was recorded at 2.25 mg/L, when the upper normal limit would normally be 0.05 mg/dL. The patient's coagulation function was continuously monitored during hospitalization (***Table 1***). Arterial blood gas analysis indicated PaO_2_ of 78 mmHg, PaCO_2_ of 38 mmHg and SaO_2_ of 94% (FiO_2_, 35%). Immunological tests and tumor markers were normal. Computed tomography pulmonary angiography (CTPA) helped to identify a massive pulmonary emboli in the bilateral pulmonary artery, right upper lung, across in the lower pulmonary artery lobe, and the left lung lower lobe pulmonary artery. Bedside TTE also highlighted a mobile mass of 5.85 cm × 4.32 cm attached to the posterior wall of the right atrium, which had partially prolapsed through the tricuspid valve during diastole. Atrial myxoma was strongly suspected. Lower limb compression venous Doppler ultrasonography revealed thrombosis in the left femoral vein (***Fig. 1***).

**Table 1:** Blood coagulation function test during the hospitalization

Date	PT (s)	INR	APTT (s)	TT (s)	FBG (g/L)	ATⅢ (%)	FDP (mg/L)	D-dimer (mg/L)
Admission	58.00	5.38	62.20	18.70	5.73	68.00	2.70	2.25
Day 3	47.30	4.35	–	–	5.58	–	–	–
Day 5	36.90	3.36	56.90	16.40	6.48	72.30	2.50	2.18
Day 7	13.00	1.13	29.50	19.20	3.26	73.70	6.10	2.13
Day 9	13.30	1.16	29.40	19.30	3.43	68.70	6.20	2.06
Day 11	13.40	1.17	32.50	21.20	2.89	63.20	8.35	1.87
Day 14	13.10	1.13	37.60	18.70	3.55	47.60	–	–
Discharge	14.80	1.29	33.50	17.10	3.26	54.60	–	1.39
APTT: activated partial thromboplastin time; ATⅢ: antithrombin Ⅲ; FDP: fibrinogen degradation product; FBG: fibrinogen; INR: international normalized ratio; PT: prothrombin time; TT: thrombin time.								

The patient received an anticoagulant consisting of 5000 IU of low-molecular-weight heparin once daily. His hemodynamics appeared stable. Further examination was refused, and therefore VTE with right atrial myxoma was tentatively diagnosed. An interdisciplinary team reviewed this case and surgery was recommended. The patient refused this treatment plan. Instead, he was discharged with a course of anticoagulants, *i.e.*, Rivaroxiban. The patient was told to take the drug twice daily (15 mg each time) for three weeks, then 20 mg once daily. He was expected to visit our outpatient clinic regularly for check-ups.

**Figure 2:**
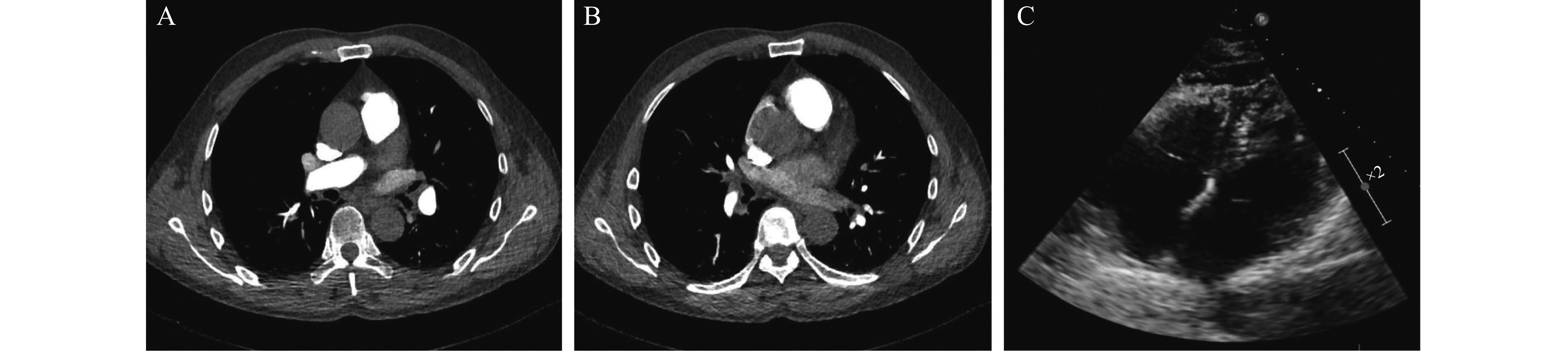
Imaging after treatment.

CTPA at three months after discharge showed no evidence of pulmonary embolism and the solid masses were no longer visible in the right heart. TTE confirmed the solid masses in the right heart had dissipated. This finding made us rethink the initial diagnosis suggesting that the solid mass was not atrial myxoma but rather a thrombus (***Fig. 2***).

## Discussion

In isolation, PE is associated with relatively high mortality. Studies have found that the all-cause mortality rate is 1.9% to 2.9% on day 7 and 4.9% to 6.6% on day 30^[5]^. Even though PE with RHTh is rare, we do know managing this comorbid disorder is complicated and early mortality is significantly increased. The mortality rate of this disease can approach 45%^[5]^. Thrombus in the right heart can arise when it sheds from DVT and travels byway of circulation to the right atrium or ventricle. Thrombus can also form "autonomously" within the right heart cavity. Risk factors of autochthonous thrombosis include pacemaker implantation, and through other surgeries, or by right atrial and ventricular diseases which alter the structure or function of the heart cavity or valve. However, these may also manifest through congenital anomalies in the heart. For instance, a 74-year-old man with acute myocardial infarction and pulmonary embolism was found to have a thrombus attached to the patent foramen ovale, partially located in right and left atria^[6]^. Of course, cancer should not be ruled out as a risk factor, especially in instances where multiple embolisms exist.

At present, RHTh can be divided into three types: type A which is free floating thrombus, and often exists in combination with critical PE, type B which is mural thrombus, and may be formed in situ in the cardiac cavity, and type C which is more rare, and attaches to part of the lumen and creates an increased risk of blocking the right atrial or ventricular outflow tract^[7]^. The solid mass observed in our patient was considered highly mobile but not floating freely in the cardiac cavity. Therefore, this case is considered to be compatible with a type C RHTh.

TTE is generally effective at diagnosing and assessing intracardiac floating thrombosis, and is useful for determining various characteristics of intracardiac masses which can inform treatment decisions. However, intracardiac mural thrombus is relatively difficult^[8]^. This helps explain why our patient's right heart mass was initially misdiagnosed as an atrial myxoma. If symptoms and circumstances permit, magnetic resonance imaging can be utilized to differentiate mural thrombus and myxoma, since the latter has heterogeneous enhancement, surface thrombus, and calcification within the mass^[9]^. Unfortunately, due to this patient's refusal, further examination was restricted.

While not common, an intracardiac mass should be considered a metastatic tumor under differential diagnosis. For instance, a right atrial mass protruding into the right ventricle arising from the inferior vena cava in a 58-year-old man was induced by the late recurrence and metastasis of renal-cell carcinoma^[10]^. However, for our patient, there was no evidence of metastasis. Researchers have found that elevated mean platelet volume is an independent predictor for left atrial thrombus^[11]^. Therefore, in addition to imaging used to detect intracardiac thrombus, the combination of several biomarkers including mean platelet volume can more accurately identify cardio-embolism.

Unfortunately, there is no consensus on the optimal treatment for concurrent PE and RHTh. To date, the majority of patients with the right heart floating thrombus in case reports have received anticoagulants, systemic thrombolytic and surgical treatment. However, if hemodynamics are instable, thrombolytic or surgical thrombectomy may be better for these patients. Under certain conditions, the minimally invasive, potentially safer option of catheter directed thrombolysis may be an alternative^[7]^. An early study has shown that thrombolytics are superior to anticoagulants alone at reducing mortality in PE with right heart floating thrombus patients^[12]^. For example, Rai *et al* presented findings from a hemodynamically stable PE with RHTh case who received anticoagulant but later died due to cardiac arrest. For this case, the treating physician believed that thrombolytic therapy would have a better outcome^[13]^. However, thrombolytics always carry the risk of secondary embolism due to ruptured thrombus and shedding.

These above treatment insights are based primarily on work with PE patients with a floating thrombus in the right heart. However, due to the extremely rare report, it remains unclear whether to apply this knowledge to patients, such as the present case. Patients with giant type C RHTh must be treated with more sensitivity and specificity to avoid poor outcomes. A previous study found that the oral anticoagulant, Apixaban is both safe and effective for a 60-year-old, female patient with left ventricular thrombus secondary to hypertrophic cardiomyopathy^[14]^. Our case further suggests that oral anticoagulants may be effective against PE with type C RHTh, if thrombolytic and surgical contraindications exist. Although, of course, we must strive to develop a database to analyze these cases retrospectively, it will take enough time to accumulate the number of cases. In the meantime, we should perhaps conduct a systematic review of the case reports to obtain further insights.

In conclusion, RHTh, especially those involving giant type C RHTh, should be carefully differentiated from cardiac tumors such as myxomas. The optimal treatment for RHTh with acute PE remains unknown. This case suggests that when the patient has type C RHTh with stable hemodynamics, anticoagulant therapy may be effective. However, further study is needed to develop best practice, although this will require interorganizational collaboration to develop a larger database that can be retrospectively analyzed.

## Acknowledgments

None.
